# Contour-Based Detection and Quantification of Tar Spot Stromata Using Red-Green-Blue (RGB) Imagery

**DOI:** 10.3389/fpls.2021.675975

**Published:** 2021-10-01

**Authors:** Da-Young Lee, Dong-Yeop Na, Carlos Góngora-Canul, Sriram Baireddy, Brenden Lane, Andres P. Cruz, Mariela Fernández-Campos, Nathan M. Kleczewski, Darcy E. P. Telenko, Stephen B. Goodwin, Edward J. Delp, C. D. Cruz

**Affiliations:** ^1^Department of Botany and Plant Pathology, Purdue University, West Lafayette, IN, United States; ^2^School of Electrical and Computer Engineering, Purdue University, West Lafayette, IN, United States; ^3^Tecnológico Nacional de México, Instituto Tecnológico de Conkal, Yucatán, Mexico; ^4^Department of Crop Science, University of Illinois, Urbana, IL, United States; ^5^U.S. Department of Agriculture-Agricultural Research Service, West Lafayette, IN, United States

**Keywords:** contour-based image segmentation, stromata detection, plant disease quantification, tar spot of corn, contour analysis

## Abstract

Quantifying symptoms of tar spot of corn has been conducted through visual-based estimations of the proportion of leaf area covered by the pathogenic structures generated by *Phyllachora maydis* (stromata). However, this traditional approach is costly in terms of time and labor, as well as prone to human subjectivity. An objective and accurate method, which is also time and labor-efficient, is of an urgent need for tar spot surveillance and high-throughput disease phenotyping. Here, we present the use of contour-based detection of fungal stromata to quantify disease intensity using Red-Green-Blue (RGB) images of tar spot-infected corn leaves. Image blocks (*n* = 1,130) generated by uniform partitioning the RGB images of leaves, were analyzed for their number of stromata by two independent, experienced human raters using ImageJ (visual estimates) and the experimental stromata contour detection algorithm (SCDA; digital measurements). Stromata count for each image block was then categorized into five classes and tested for the agreement of human raters and SCDA using Cohen's weighted kappa coefficient (κ). Adequate agreements of stromata counts were observed for each of the human raters to SCDA (κ = 0.83) and between the two human raters (κ = 0.95). Moreover, the SCDA was able to recognize “true stromata,” but to a lesser extent than human raters (average median recall = 90.5%, precision = 89.7%, and Dice = 88.3%). Furthermore, we tracked tar spot development throughout six time points using SCDA and we obtained high agreement between area under the disease progress curve (AUDPC) shared by visual disease severity and SCDA. Our results indicate the potential utility of SCDA in quantifying stromata using RGB images, complementing the traditional human, visual-based disease severity estimations, and serve as a foundation in building an accurate, high-throughput pipeline for the scoring of tar spot symptoms.

## Introduction

Plant disease assessments are conducted to quantitatively measure the amount of disease (intensity) in a host population (Campbell and Madden, 1990; Nutter et al., 1993). Nevertheless, plant disease epidemics occur from the interaction of both host and pathogen populations with the environment in space and time. Therefore, symptoms and signs of diseases are expected to be directly proportional to the size of the pathogen population (Nutter et al., 2006; Groves et al., 2020). Hence, disease assessments can be conducted by estimations or measurements of the extent of disease symptoms or signs of the pathogen (e.g., number of spores, sclerotia, and stromata) per unit area of the plant sampled (Nutter, 1997, 1999).

Despite the significant role that human vision-based disease evaluation has played in the advancement of plant pathology, the accuracy of this traditional way of disease estimation has continuously been questioned due to the “human factor” that is part of the endeavor (Sherwood et al., 1983; Shokes et al., 1987; Nutter and Schultz, 1995; Nutter and Esker, 2006; Nutter et al., 2006). To address these problems, digital imagery-based disease phenotyping has extensively been explored during the past decade for its potential in mitigating the limitations of human visual-based disease estimates (Mahlein, 2016; Simko et al., 2017; Bock et al., 2020). The feasibility of digital image processing has been assessed and is widely used for plant disease quantification (Tucker and Chakraborty, 1997; Bock et al., 2008; Gongora-Canul et al., 2020). The utility of Red-Green-Blue (RGB) image-based processing and deep learning have shown a great promise for the recognition and quantification of various plant diseases (Lamari, 2002; Bardsley and Ngugi, 2013; Stewart and McDonald, 2014; Ngugi et al., 2021).

Tar spot, caused by *Phyllachora maydis* Maubl., is a fungal disease of corn that is endemic to Mexico and to various countries in Central and South America (Maublanc, 1903). The disease has established itself across the northern US since 2015 (Ruhl et al., 2016; McCoy et al., 2018; Dalla Lana et al., 2019; Kleczewski et al., 2019; Mueller et al., 2020; Valle-Torres et al., 2020) resulting in ~$840 million in losses during 2018–2019 (Crop Protection Network, 2021). Phenotyping and surveillance of tar spot have been performed through human visual assessments of disease severity based on the detection of pathogenic structures called stromata. These black-brown, semi-circular growths are produced as a result of *P*. *maydis* infection and are embedded in host tissue and can be observed across leaf surfaces and other tissues (Liu, 1973; Hock et al., 1995; Carson, 1999; Kleczewski et al., 2019; Valle-Torres et al., 2020). In addition, *P*. *maydis* can produce ascospores in sexual structures embedded in the stromata, acting as inocula (Kleczewski et al., 2019; Valle-Torres et al., 2020). Therefore, the proportion of stromata relative to the area of the corn leaf has been estimated to reflect tar spot severity. Moreover, since stromata can also serve as a measure of pathogen colonization of infected plant tissue, the number of stromata per unit area of the infected corn leaf serves as an important measure of tar spot severity.

Despite the growing importance of tar spot, a standardized, objective method capable of high-throughput assessments of its symptoms is not available. Interpretation of the symptom intensity data collected for tar spot is essential to guide disease-management decisions (Bock et al., 2010). Hence, selecting the method suited to accurately represent the intensity of a disease of interest is crucial (Campbell and Madden, 1990; Gaunt, 1995). The objectives of our study were to (i) develop a tar spot stromata contour detection algorithm (SCDA) using RGB images of tar spot-infected corn leaves; (ii) assess the performance of the SCDA by comparing the numbers and locations of the stromata to those determined by two independent human raters (reference data), and evaluate its feasibility in tracking tar spot disease development in the field by comparing it to human visual disease estimations and an alternative machine learning-based approach. A reliable, accurate, high-throughput method for tar spot assessment will benefit plant disease modeling, epidemiology, and resistance screening.

## Materials and Methods

### Leaf Sample Collection and Red-Green-Blue Image Acquisition

Two datasets were generated and used in this study. The first consisted of tar spot-infected maize leaves randomly collected from a field experiment site established at the University of Illinois South Farm in Urbana-Champaign, Illinois. Fresh leaf samples were pressed to flatten out the leaf edges and brought back to our laboratory. RGB images were acquired using a Canon E.O.S. 6D full-frame 20.2 MP DSLR camera body and a Canon E.F. 50 mm f/1.8 S.T.M. lens. A 30 × 70 cm cardboard panel covered by a synthetic blue fleece fabric was laid out on a flat surface as a background for all leaf samples to facilitate effective background removal during the image preprocessing step. Photographs were taken in .jpg format and then converted to .png format. The second dataset (Dataset B) contained RGB images of maize leaves acquired during the summer of 2020 at the Pinney Purdue Agricultural Center (PPAC) in Wanatah, Indiana. A tar spot fungicide trial was established at PPAC with a randomized complete block design. The fungicide treatments were randomly assigned into blocks and four replications were established. We selected two plots per replication, an untreated and experimental setup, of which from preliminary results, we have identified the high efficacy of the experimental setup [Headline AMP, 10 fl oz + Preference (NIS), 0.25% v/v] in managing tar spot. Each plot consisted of four rows of which the middle two rows were used to collect both image- and visual-rating data. A total of five maize plants in the middle two rows were selected in a zig-zag manner, wherein two leaves from the middle canopy of each selected maize plant were marked and tagged to track tar spot development at different time points. The RGB images of the leaves were collected approximately at weekly intervals over six time points during 2020: August 25, September 3, 10, 15, 22, and 29. Collectively, 466 RGB images of selected maize leaves were then used as input and analyzed using SCDA and a maskRegion-based convolutional neural network (maskR-CNN) approach (unpublished data).

### Generation of “Image Blocks”

Quantification of disease severity can be done at the scale of plant organs (e.g., the stems and the leaves) or in quadrats (Bock et al., 2010). In our study, we partitioned six tar spot-infected leaf RGB images (samples A–F) into uniformly-sized (400 × 400 pixels) squares or “image blocks” which contained different regions of the corn leaf with varying numbers of stromata. A total of 1,130 image blocks (Sample A = 202 blocks, Sample B = 196 blocks, Sample C = 193 blocks, Sample D = 131 blocks, Sample E = 217 blocks, and Sample F= 191 blocks) were provided to the human raters for software-aided, visual assessment and also used as input for stromata contour analyses.

### Reference (Human Visual-Based) Data Tar Spot Disease Quantification

In this study, we utilized the terms, “estimate” and “measurement” to refer to assessments conducted by human rater (visual) and the SCDA, respectively. To generate reference ground truth for Dataset A, two human raters with experience in tar-spot disease estimations were employed to generate reference data to assess the performance of the SCDA. Human raters analyzed the number of stromata for each image block with the help of the point toolbox (yellow, cross-shaped markers) provided by Fiji (Image J; Schindelin et al., 2012), wherein raters clicked on the center of all structures perceived as stromata. Furthermore, the reference disease severity data for Dataset B was in the form of estimated percentage leaf area covered by stromata. Estimations were done for the lower, middle, and upper canopy per experiment plot. The prominent ear leaf was considered leaf 0 (L0). Leaves below or above L0 were identified with signs “–,” and “+,” respectively. The lower canopy was from L-3 to the lowest leaf (L - n), mid-canopy from L-2 to L + 1, and the upper canopy from L + 2 to the flag leaf (L + n).

### Assessing the Agreement Between SCDA and Human Raters

To measure the agreement of stromata counts for all the image blocks analyzed by two independent raters as well as between those of the raters and the SCDA, weighted Cohen's kappa coefficient (κ) was used. Cohen's kappa is a metric to assess the agreement between two raters, i.e., the two raters either agree in their rating or disagree. However, it does not quantify the extent of disagreement. Weighted Cohen's kappa with a modification to Cohen's kappa can resolve this issue, using predefined weights that measure the degree of disagreement between the two raters; the higher the disagreement, the higher is the weight. For instance, let (1) *n* be the total number of subjects, (2) *n*_*i*_ be the number of subjects for which rater A chooses category *i*, (3) *m*_*j*_ be the number of subjects for which rater B selects category *j*, and (4) *n*_*i,j*_ be the number of subjects for which raters A and B choose categories *i* and *j* at the same time, respectively. Defining *p*_*i*_ = *n*_*i*_/*n*, *q*_*i*_ = *m*_*j*_/*n*, and *p*_*i,j*_ = *n*_*i,j*_/*n*, one can calculate the weighted Cohen's kappa (Bakeman and Gottman, 1997) by:


$$\begin{array}{l} \kappa =1-\left(\underset{i,j}{\sum }w_{i,j}p_{i,j}\right)/\left(\underset{i,j}{\sum }w_{i,j}e_{i,j}\right)\;, \end{array}$$

where *e*_*i,j*_ = *p*_*i*_*q*_*i*_ are the expected probabilities and *w*_*i,j*_ are the weights.

The collected nominal data were then classified into five categories (Classes 1–5) and the resulting ordinal categorical data were used to calculate Cohen's weighted kappa index for the agreement between the ordinal data. The five groups were delimited as: Class 1 (0 to 2 stromata); Class 2 (3 to 9 stromata); Class 3 (10 to 20 stromata); Class 4 (21 to 45 stromata); and Class 5 (>46 stromata). The kappa coefficient ranges from −1 to 1, wherein the value of −1 indicates complete disagreement (poor agreement), 0 indicates agreement by chance, and 1 indicates perfect agreement. The strength of agreement for positive kappa values can be further categorized as slight (0.01–0.20), fair (0.21–0.40), moderate (0.41–0.60), substantial (0.61–0.80), or nearly perfect (0.81–0.99) (Landis and Koch, 1977; Shoukri et al., 1999).

### Assessing the Performance of the Tar-Spot SCDA to Recognize Stromata Compared to Human Raters

To assess the performance of the SCDA in recognizing stromata at the human rater level, we utilized the same image blocks which were previously used to quantify the number of stromata. Using ImageJ, human raters labeled the centers of the stromata by using yellow cross markers in all image blocks and saved the labeled images. Then, using MATLAB, these labeled images were loaded and converted into a binary mask by the following threshold condition of detecting all pixels colored in yellow:


$$\begin{array}{l} {\left[{\bar{\text{T}}}_{h}\right]}_{i,j}=\{\begin{array}{cc} true\;or\;1, & \;if\;{\left[\bar{\text{R}}\right]}_{i,j}={\left[\bar{\text{G}}\right]}_{i,j}=255\;and\;{\left[\bar{\text{B}}\right]}_{i,j}=0\\ false\;or\;0, & \;otherwise \end{array} \end{array}$$

where ${\bar{\text{T}}}_{h}$ is the resulting binary mask that encodes spatial locations of stromata detected by the human raters. The binary mask ${\bar{\text{T}}}_{h}$ was then compared to the binary mask produced by the SCDA algorithm, denoted by ${\bar{\text{T}}}_{a}$. If an isolated region having 1 in ${\bar{\text{T}}}_{a}$ spatially overlaps with a region having 1 in ${\bar{\text{T}}}_{h}$, this region or the corresponding stroma had spatial coincidence using both human and SCDA methods; it was considered to be a true positive. This task was repeated for all isolated regions having 1 in ${\bar{\text{T}}}_{a}$. On the other hand, if an isolated region having 1 in ${\bar{\text{T}}}_{a}$ did not overlap with any region having 1 in ${\bar{\text{T}}}_{h}$, it was regarded as a false positive. Finally, if an isolated region having 1 in ${\bar{\text{T}}}_{h}$ did not overlap with any region having 1 in ${\bar{\text{T}}}_{a}$, it was considered as a true negative. After processing all the image blocks, precision, recall, and Dice coefficient metrics were calculated. Precision measures the correctly identified positive cases among all predicted positive cases. Thus, it is a useful figure of merit to observe whether the cost of false positives is high. Recall measures the correctly identified positive cases against all the actual positive cases and is an important metric when the cost of false negatives is high. Dice coefficient (or F1-score) is proportional to the harmonic mean of precision and recall and is calculated to assess the spatial overlap shared by the ground truth (i.e., human raters A or B) and the SCDA for a comprehensive measure of the incorrectly classified cases.

Precision, recall, and Dice coefficients measured in percentage (%) are defined as follows:


$$\begin{array}{l} Precision\;\left(\%\right)=\frac{TP}{TP+FP}\times \;100,\\ \;Recall\;\left(\%\right)=\frac{TP}{TP+FN}\times 100,\\ \;Dice\;\left(\%\right)=2\times \frac{Precision\times Recall}{Precision+Recall}\times 100\\ \;=\frac{2\times TP}{2\times TP+FN+FP}\times 100, \end{array}$$

where TP = true positive, FP = false positive, and FN = false negative. TP is defined by the number of true stromata correctly detected by the SCDA, FN is the number of true stromata undetected, and FP is the number of wrong stromata detected by the SCDA. The results of all blocks of each sample leaf were transformed into a histogram, showing the probability density vs. percentage for precision, recall, and Dice coefficient metrics. Note that the total area of each histogram (i.e., Riemann sum) is supposed to be equal where the width of the bin is chosen as 4%.

### Quantification of Tar Spot Intensity Using Mask R-CNN

Parallel to SCDA, mask R-CNN approach was used as a deep learning approach to detect tar spot stromata. The output of this approach provided stromata counts and the proportion of leaf area covered by stromata, which were then statistically analyzed for a side-by-side evaluation of its performance and that of SCDA and agreement with the visual data.

### Assessing the Agreement Between Reference Visual Data to the SCDA and a Mask R-CNN Approach

The area under disease progress curve (AUDPC) using visual severity estimation of tar spot at different canopy levels was used as reference data to measure the agreement with digital counts of stromata and the area occupied by stromata measured by the two algorithms. Accuracy, precision, and bias of digital disease measurements (Nutter et al., 1991; Madden et al., 2007) were evaluated. However, before measuring the agreement, AUDPC values from visual and the algorithm data were matched with similar scale values according to the maximum and minimum values. Accuracy is a product of precision and bias (Nita et al., 2003; Madden et al., 2007). Accuracy was calculated with Lin's concordance correlation coefficient (ρ_*c*_) which measures the variation of data from a concordance line, a 1:1 line with an intercept of zero and a slope of one (Lin, 1989; Nita et al., 2003; Bock et al., 2010). To obtain ρ_*c*_, we used the equation ρ_*c*_ = *r* × *C*_*b*_, where *r* represents the correlation coefficient as the measurement of precision (r = 1 perfectly straight line), while *C*_*b*_ as the measurement of bias (closeness of best fit line to the concordance line; *C*_*b*_ = 1 indicates no bias). The *C*_*b*_ was calculated with the equation


$$\begin{array}{l} C_{b}=2/\left[u^{2}+\nu \;+\left(1/\nu \right)\right], \end{array}$$

where ν = (σ_1_/σ_2_*)* indicated scale shift or difference in the slope of the concordance and best-fit lines (ν = 1 for equal slopes), and $u=[(\mu_{1}-\mu_{2})/\sqrt{\left(\sigma_{1}\times \sigma_{2})\right]}$ corresponds to location shift or differences in height (*u* = 0 for equal intercepts). Furthermore, μ_1_ and μ_2_ are the means of measured values/digital disease measurement and true values/visual disease estimates, while σ_1_ and σ_2_ are the standard deviations of these values calculated based on maximum-likelihood estimates (Nita et al., 2003; Madden et al., 2007). The analysis was performed using PROG REG ALL procedure on SAS (SAS Institute, Cary NC), based on the macro statement developed by Lawrence Lin and verified by Min Yang (Lin et al., 2002).

## Theory and Calculation

### Image Pre-processing

#### Background Removal

**Figure 1:**
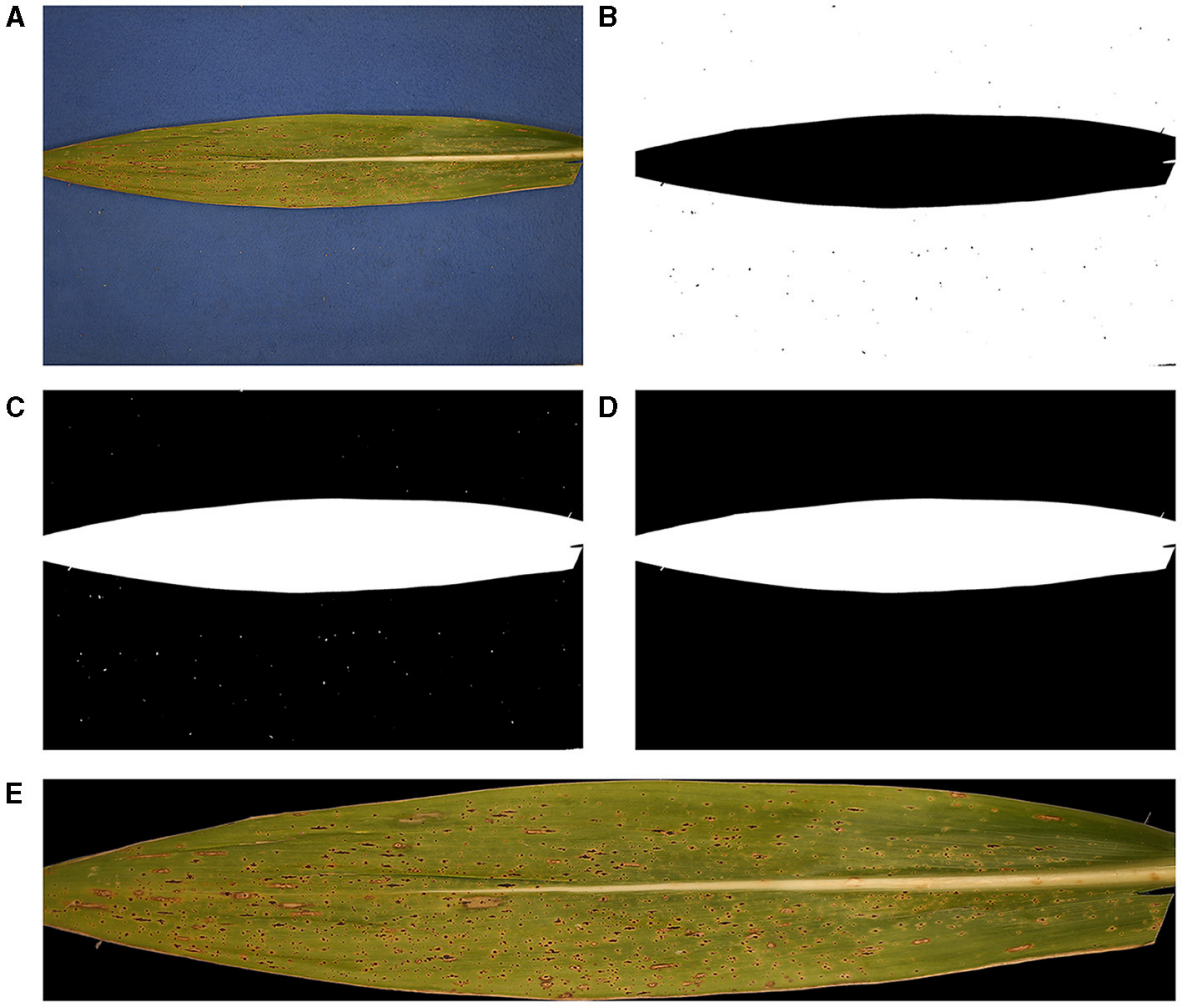
Pre-processing of original tar spot-infected leaf images. **(A)** Original RGB image, **(B)** binary image after thresholding, **(C)** complement of the binary image, **(D)** resultant mask, and **(E)** cropped sample image after isolating the region of interest (RoI) (leaf image without background).

The images in Dataset A had a blue background panel behind the corn leaf for easier background removal. Since the color properties of the corn and the blue panel can be distinguished by a simple thresholding, one can obtain the region of interest (RoI) of the diseased corn leaf easily. The SCDA pipeline starts first by reading the input RGB image, denoted by *I*. Its red, green, and blue channel matrices are represented by $\bar{\text{R}}$, $\bar{\text{G}}$, and $\bar{\text{B}}$, respectively. The RGB images containing the diseased corn leaf with a blue panel as background were utilized as input, as shown in Figure 1A. Thresholding color values isolated the region of interest, or the corn leaf *via* the following conditions:


$$\begin{array}{l} {\left[\bar{\text{T}}\right]}_{i,j}=\{\begin{array}{l} 1,\;if\;{\left[\bar{\text{B}}\right]}_{i,j}>{\left[\bar{\text{R}}\right]}_{i,j}\;and\;{\left[\bar{\text{B}}\right]}_{i,j}>{\left[\bar{\text{G}}\right]}_{i,j}\\ 0,\;otherwise \end{array} \end{array}$$

for all pixels, i.e., *i* = 1, 2, ⋯ , *N*_*r*_ and *j* = 1, 2, ⋯ , *N*_*c*_ where *N*_*r*_ and *N*_*c*_ are the number of horizontal and vertical pixels. Note that $\bar{\text{T}}$ is the resulting binary image mask after thresholding.

An example of the resulting binary image is depicted in Figure 1B, in which pixels having value 1 are visualized by white, whereas the other pixels are visualized by black. Next, we complemented the binary image, i.e., values at all pixels are reversed (Figure 1C), and salt-and-pepper noise was removed by performing CCC or ρ_*c*_ erosion and dilation, which are deleting and adding of pixels to the boundary of an original object, respectively, depending on the size and shape of the structuring element. The resulting mask, denoted by $\bar{\text{M}}$, is illustrated in Figure 1D. Finally, RoI can be obtained by performing the Hadamard product of each channel matrix ($\bar{\text{R}}$, $\bar{\text{G}}$, or $\bar{\text{B}}$) of the original RGB image and $\bar{\text{M}}$, i.e.,


$$\begin{array}{l} {\bar{\text{R}}}_{ROI}=\bar{\text{R}}◦\bar{\text{M}},\;{\bar{\text{G}}}_{ROI}=\bar{\text{G}}◦\bar{\text{M}},\;{\bar{\text{B}}}_{ROI}=\bar{\text{B}}◦\bar{\text{M}}. \end{array}$$

Furthermore, for computational efficiency, non-RoI regions were discarded by introducing a window (rectangular box) that only contained RoI. This can be done by measuring the size of the RoI, i.e., minimum and maximum indices of rows and minimum and maximum indices of columns, denoted by, *r*_min, *roi*_, *r*_max, *roi*_, *c*_min, *roi*_, *c*_max, *roi*_, respectively. The final RGB image to be analyzed is illustrated in Figure 1E.

In contrast to Dataset A, which consisted of images acquired under controlled lighting conditions (indoor), Dataset B comprised images which were acquired under natural lighting conditions with varied focus. An advanced background remover was required to correctly isolate the RoI; however, the development of such a tool was beyond the scope of this study. Instead, we utilized a commercial artificial-intelligence-based, background image remover, Clipping Magic, which enabled us to process 466 RGB images of maize leaves collected in the field with arbitrary background. The processing time for each RGB image was <10 s. Subsequent procedures for detecting tar spot stromata were the same as those used for the previous analysis of Dataset A, where the image is then partitioned into image blocks.

#### Homogenization of Inhomogeneous Brightness of RGB Images

**Figure 2:**
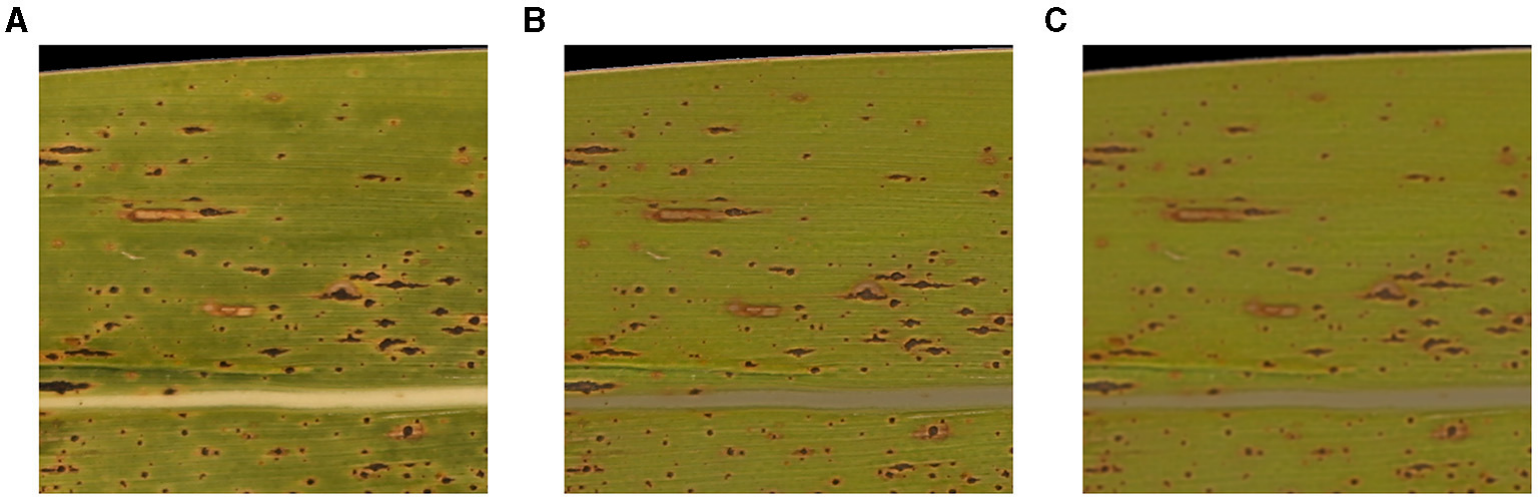
Homogenization and Gaussian filtering of image blocks. **(A)** Pre-homogenization, **(B)** post-homogenization, and **(C)** post-filtering.

Due to the prevailing conditions, when taking pictures of corn leaves, such as weather and time, raw RGB images often have inhomogeneity that can degrade the accuracy of detecting tar spot stromata. An example of an image block imposed by the intensity inhomogeneity of a raw image is illustrated in Figure 2A. To homogenize the brightness of the image blocks so that the false detection rate of tar spot stromata can be minimized, MATLAB built-in function imflatfield() was utilized (Figure 2B). Moreover, to prevent false-positive detections, such as the salt-and-pepper noises, MATLAB built-in function imgaussfilt() was used to apply the Gaussian filter, which blurred the input RGB image and reduced its resolution (Figure 2C). This approach will also improve the computation speed with the use of fewer contours.

#### Converting the RGB Images Into Grayscale Images

**Figure 3:**
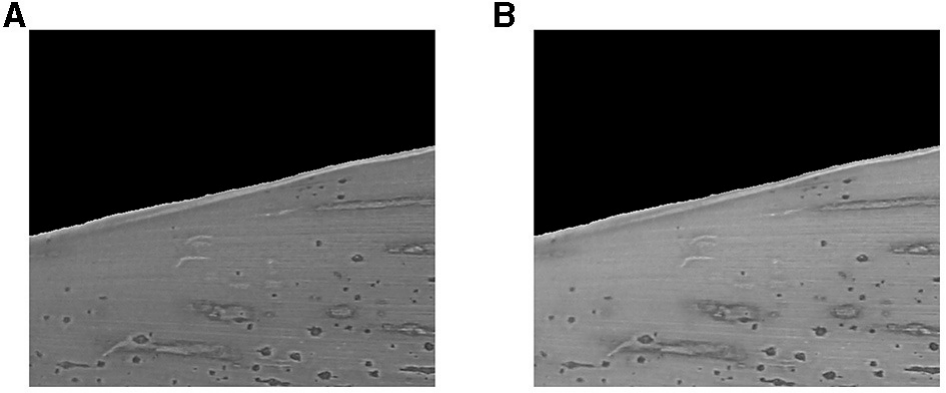
Normalized grayscale images $\bar{Q}$ using the **(A)** average method and **(B)** weighting method.

Each input RGB image (three channels) was converted into a grayscale image (one channel) to generate contour lines. The naïve average method (Niblack, 1986; Solomon and Breckon, 2011),


$$\begin{array}{l} {\left[\bar{\text{P}}\right]}_{i,j}=0.3333{\left[\bar{\text{R}}\right]}_{i,j}+0.3333{\left[\bar{\text{G}}\right]}_{i,j}+0.3333{\left[\bar{\text{B}}\;\right]}_{i,j} \end{array}$$

where $\bar{\text{P}}$ is the resultant grayscale image (Figure 3A), generally produces a darker grayscale image. Consequently, the resulting image may lose the distinct patterns manifested by the stromata structure and may degrade the contrast between the stromata patterns and the surrounding regions (leaf area). To avoid this problem, we used the weighted (or luminosity) method, which combines RGB colors with different weighting factors (Figure 3B). The weighted method resolves the issue mentioned above, given by


$$\begin{array}{l} {\left[\bar{\text{P}}\right]}_{i,j}=0.2989{\left[\bar{\text{R}}\right]}_{i,j}+0.5870{\left[\bar{\text{G}}\right]}_{i,j}+0.1140{\left[\bar{\text{B}}\right]}_{i,j}. \end{array}$$

Then, we can normalize this as


$$\begin{array}{l} {\left[\bar{\text{Q}}\right]}_{i,j}=\frac{{\left[\bar{\text{P}}\right]}_{i,j}-min\left(\bar{\text{P}}\right)}{max\left(\bar{\text{P}}\right)-min\left(\bar{\text{P}}\right)} \end{array}$$

where $max\left(\bar{\text{P}}\right)$ and $min\left(\bar{\text{P}}\right)$ are maximum and minimum values of elements in the matrix $\bar{\text{P}}$. As a result, one can obtain the resulting normalized grayscale image $\bar{\text{Q}}$ in which elements range from 0 to 1 with double data type.

### Generating Contour Lines to Detect Tar Spot Stromata

The choice of contour analysis, for the detection of tart spot stromata, was motivated by the morphology of the pathogen structure (stromata), characterized by the protrusion of black and semi-circular regions on the leaf surface, leading to an elevated and rough topology (Valle-Torres et al., 2020). Suppose that a scalar function is defined on the 2-dimensional Cartesian coordinate system, denoted by *f*(*x, y*). The function value takes a scalar number at a given position (*x, y*). A contour line (isoline) is made of a set of points connected so that their function values are equal. Different contour lines represent another set of points having different function values. Here, the function values of contour lines correspond to values of the grayscale image at a given pixel obtained in the previous step. Thus, contour lines describe the brightness of the pixels in the image. Note that contour lines can be generated by using the built-in function contour() in MATLAB.

**Figure 4:**
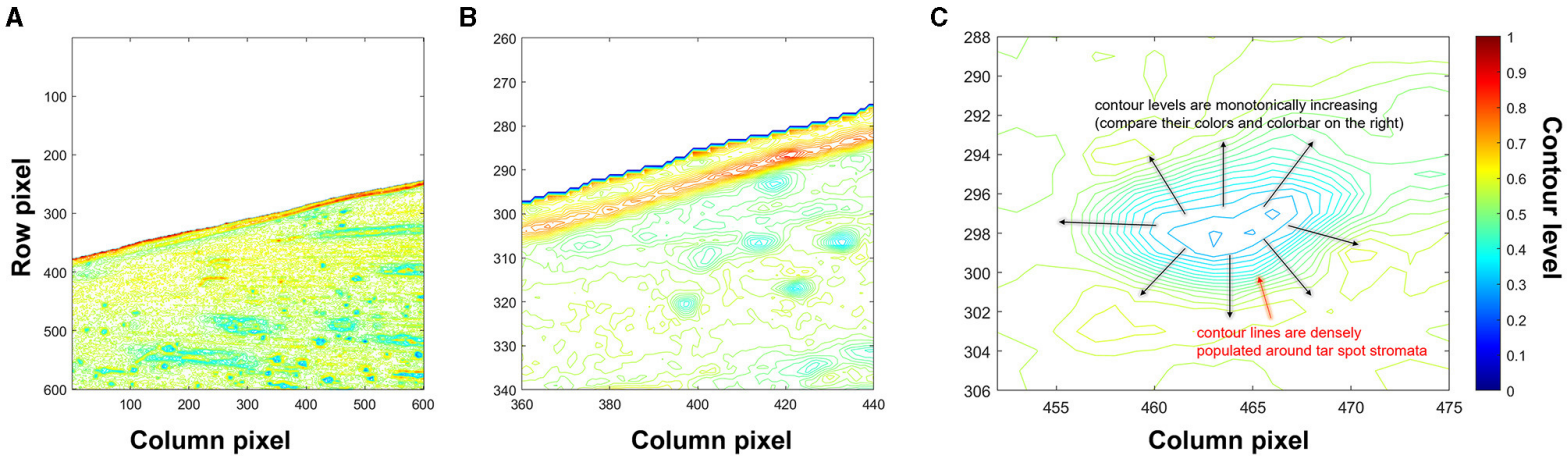
Contour lines generated from a normalized grayscale image block. **(A)** All contour lines, **(B)** zoomed-in view of contour lines where columns from 360 to 440 and rows from 260 to 340, and **(C)** distinct pattern of contour lines of stromata.

Figures 4A,B illustrate contour lines for a representative image block. Note that the color of each contour line represents the brightness of the normalized grayscale image. Brightness can be thought similarly as heights of the contour lines on a map. Furthermore, Figure 4C illustrates contour lines of tar spot stromata in a zoomed window at rows from 452 to 475 and columns from 288 to 306, wherein the contour lines near a stroma show distinct patterns of densely populated contour lines that monotonically increase the brightness levels from the center of the stroma toward the outer boundary of the structure. This feature facilitated the search for sets of contour lines showing such patterns, which were predicted to delineate tar spot stromata.

### Feature Extraction: Identification of Tar Spot Stromata and Local Contour Analysis

Consider a set of points, denoted by $\left(x_{j}^{\left(i\right)},y_{j}^{\left(i\right)}\right),$ which represents the *j*-th point of the *i*-th contour line. The degree of circularity of a polygon formed by the *i*-th contour line is calculated and the area can be evaluated by a MATLAB built-in function polyarea(). The center point of the *i*-th contour line, denoted by $\left(x_{cent}^{\left(i\right)},y_{cent}^{\left(i\right)}\right)$, of the contour line can be calculated by averaging all points consisting of the *i*-th contour line. The degree of circularity, denoted by *g*_*circle*_, can be evaluated by


$$\begin{array}{l} g_{circle}=d_{\min}/d_{\max} \end{array}$$

where *d*_min_ and *d*_max_ are minimum and maximum radii, i.e., distances between each point and the center point of the contour line, which can be written by = min(**d**) and = max(**d**) where,


$$\begin{array}{l} {\left[\text{d}\right]}_{j}=\sqrt{{\left(x_{cent}^{\left(i\right)}-x_{j}^{\left(i\right)}\right)}^{2}+{\left(y_{cent}^{\left(i\right)}-y_{j}^{\left(i\right)}\right)}^{2}}. \end{array}$$

Note that *g*_*circle*_ has the range of 0 ≤ *g*_*circle*_ ≤ 1; in other words, the higher the *g*_*circle*_value, the more circular the shape of a contour will be (i.e., *g*_*circle*_ = 1 for a perfect circle).

**Figure 5:**
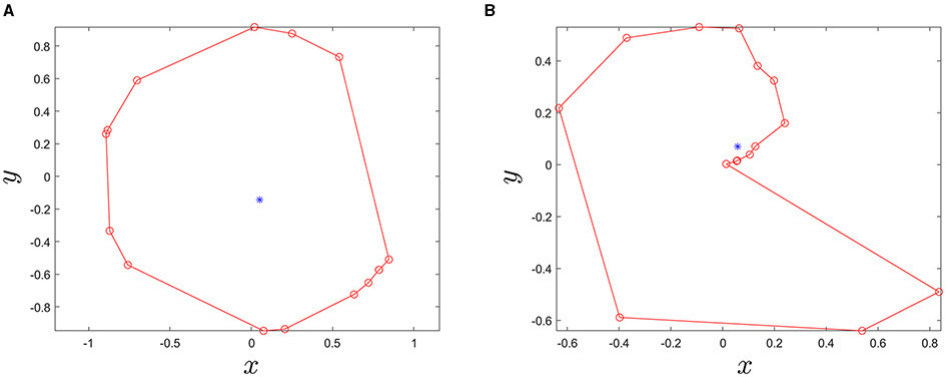
Two example contour lines. Red circle markers are points of each contour line and the blue dots indicate the center point of polygons **(A)** *g*_*circle*_ = 0.7606, *area* = 2.3821 and **(B)** *g*_*circle*_ = 0.0566, and area = 0.9515.

Figure 5 compares the degree of circularity of two example contour lines. Since the shape of tar spot stromata is often semi-circular or circular, the contour lines with very low values of *g*_*circle*_ can be discarded. For the contour lines which overlap, a contour line satisfying the following conditions was regarded as a tar spot stroma and searched: (1) the contour line must completely enclose more than *N*_*ct*_ number of smaller sub-contour lines, (2) the ratio of areas of the nearest sub-contour lines should be less than *r*_*ad*_ (threshold value), and (3) contour levels should be monotonically decreasing from the largest to the smallest contour lines.

**Figure 6:**
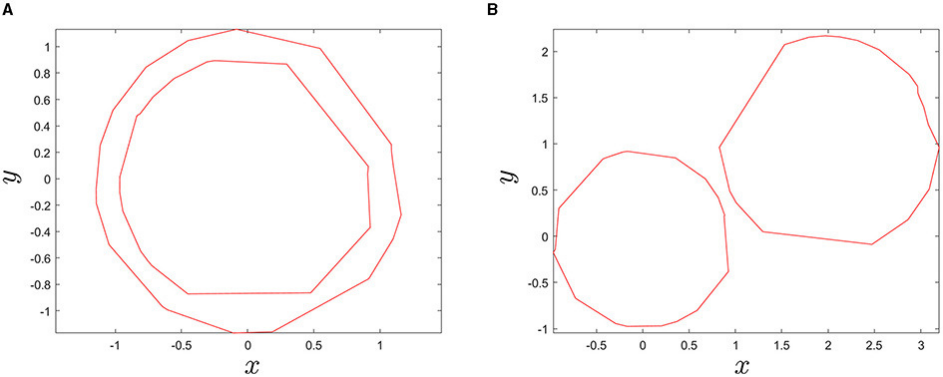
Illustration showing instances when two given contour lines are either **(A)** enclosed or **(B)** not enclosed using the MATLAB built-in function overlaps().

**Figure 7:**
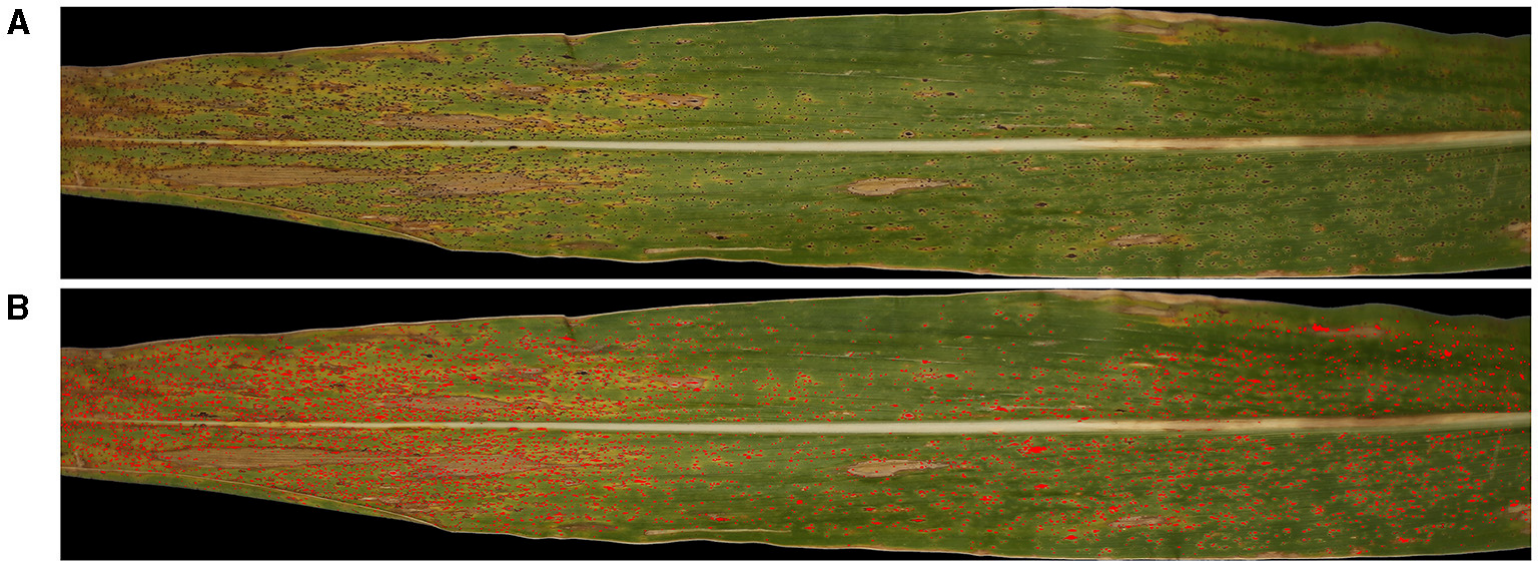
Illustration, showing the **(A)** original Red-Green-Blue (RGB) image input (top) and the **(B)** resulting image after detection of stromata colored in red using the SCDA (bottom).

Consequently, the largest contour line found would correspond to the boundary of the tar spot stroma and its interior region becomes the area of the stroma. Thus, the largest contour line that we found can be called a stroma-boundary-contour line. To check enclosedness between two contour lines, built-in function overlaps() in MATLAB was used (Figure 6). Finally, the searching algorithm was repeatedly performed to find all stroma-boundary-contour lines for all blocks in a sample corn leaf in Figures 7A,B.

## Results

### Agreement Between the Stromata Contour Detection Algorithm and Human Raters A and B With Respect to Detection and Quantification of Stromata

**Figure 8:**
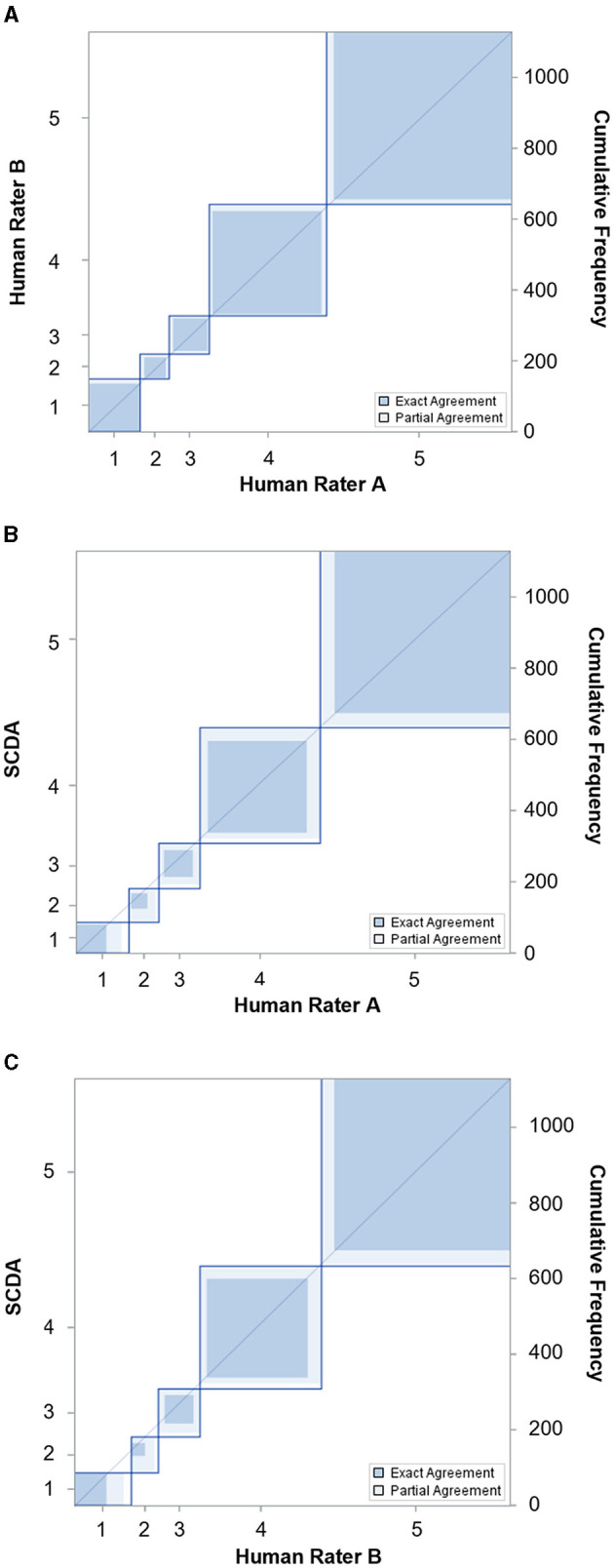
Agreement charts reflecting the agreement of tar spot stromata counts (classes 1 to 5) between **(A)** two human raters, **(B)** human rater A and SCDA, and **(C)** human rater B and SCDA after kappa analysis.

A total of 1,130 of image blocks was evaluated for the number of stromata by two independent human raters and by the stromata contour detection algorithm (SCDA). The capability of the SCDA to recognize Classes 1, 2, and 3 was slightly biased compared to the human raters, but the kappa strengths of the agreement between SCDA and human raters A (κ = 0.83) and B (κ = 0.83) were classified as nearly perfect and identical to the strength of agreement observed between human raters A and B (κ = 0.95) (Figure 8; Table 1).

**Table 1:** Agreement of two independent raters and SCDA according to Cohen's weighted kappa and associated 95% confidence levels (CI).

**Comparisons**	**Weighted kappa**			
	**κ**	**Z-statistic**	**CI**	**Pr > F**
Human rater A vs. B	0.9494	44.4357	0.9384–0.9605	<0.0001
Human rater A vs. SCDA	0.8297	39.7888	0.8075–0.8518	<0.0001
Human rater B vs. SCDA	0.8283	39.7702	0.8060–0.8505	<0.0001

### Assessment of the Ability of SCDA to Accurately Detect Stromata Compared to Human Raters

**Figure 9:**
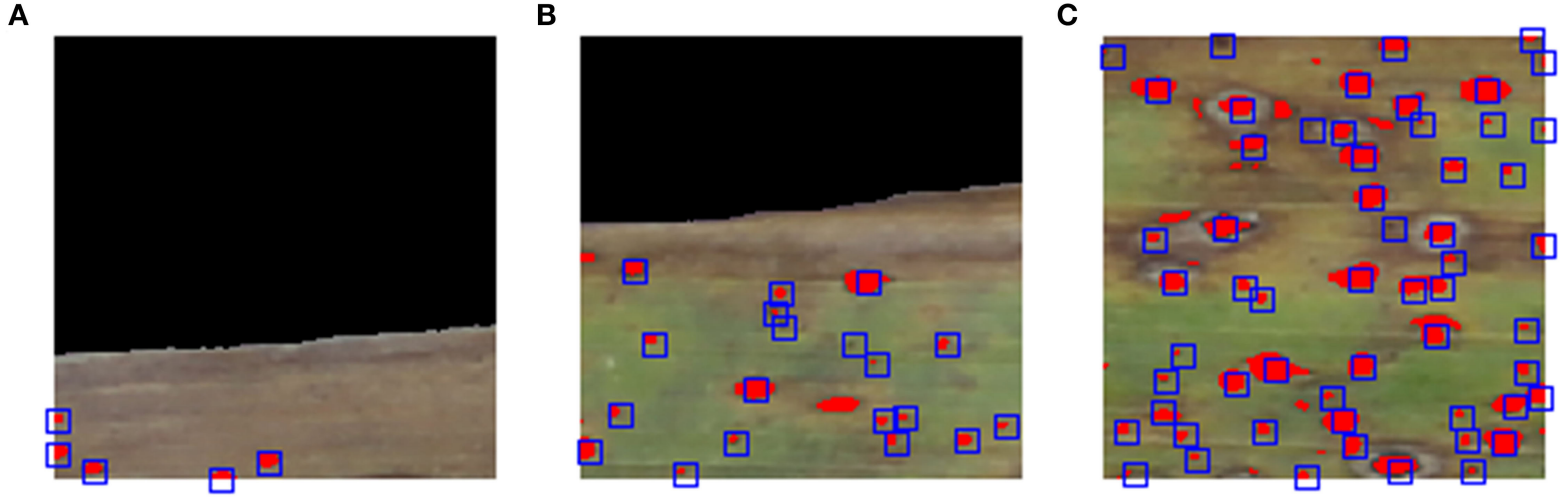
Overview of evaluating spatial overlap of stromata recognized by human rater A and contour analysis. Illustration of evaluating random image blocks **(A–C)**, showing infected leaf image blocks with varying numbers of stromata (increasing frequency of stromata from left to right). Red blobs represent the area of stromata detected by contour analysis while the center of blue squares signifies the point which the human raters identified to be stromata.

The higher concordance correlations between the numbers of stromata detected by the SCDA vs. human raters is not enough to evaluate its performance. For more accurate validation purposes, the coincidence rate for each stroma detected was measured both by the algorithm and human raters. Figure 9 depicts the coincidence measurement (the present algorithm vs. human rater A) for three example blocks. Image blocks were chosen randomly while showing infected leaf image sections with varying numbers of stromata (increasing frequencies of stromata, from left to right).

**Figure 10:**
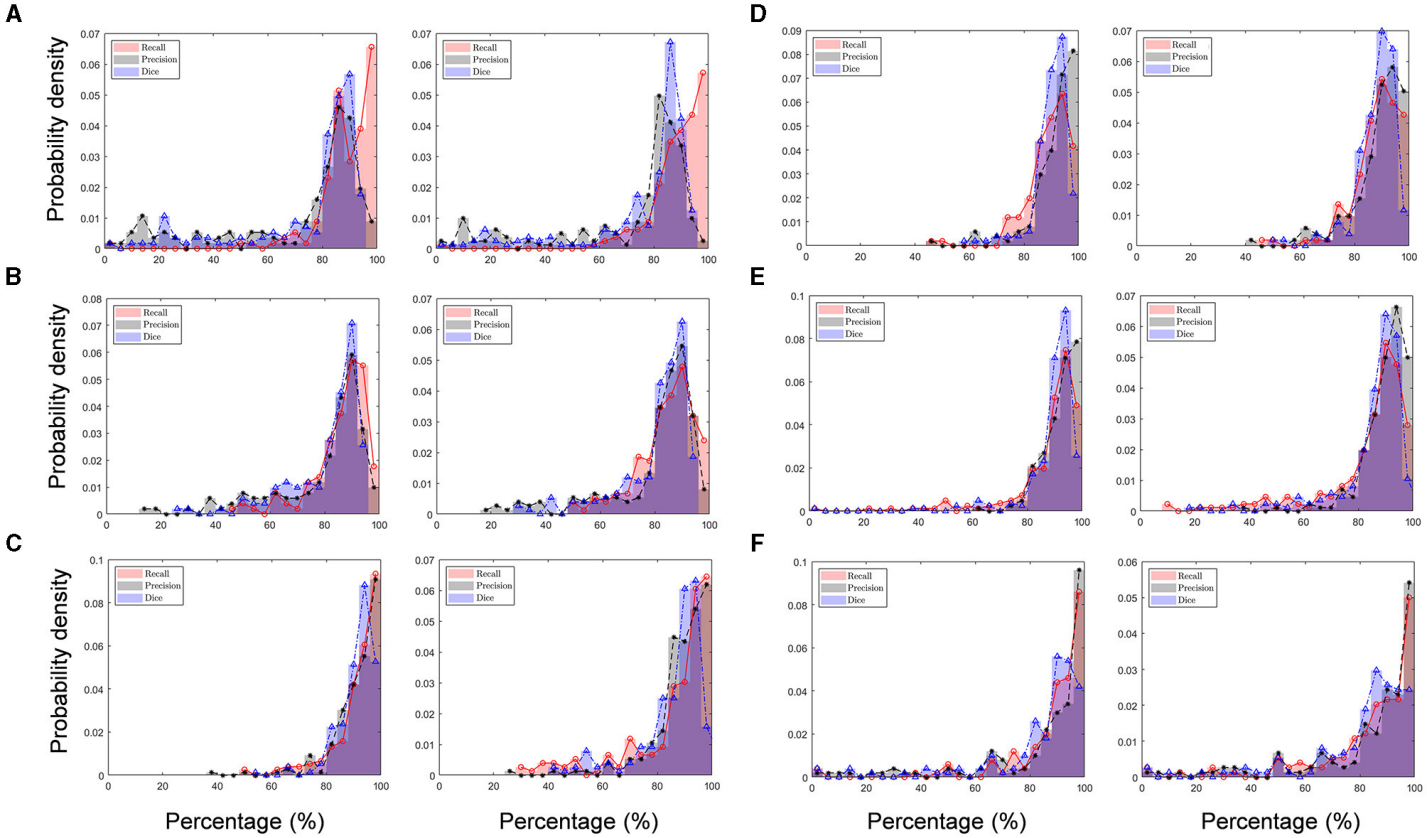
Validation of performance (precision, recall, and Dice coefficient) of the stromata contour algorithm (SCDA) in recognizing “true stromata” compared to human rater A (left panels) and human rater B (right panels) on **(A)** Sample A, **(B)** Sample B, **(C)** Sample C, **(D)** Sample D, **(E)** Sample E, **(F)** and Sample F.

The mean and median values of recall for all the image blocks analyzed by both human raters A and B ranged from 83.3 to 91.7% (average: 87.1%) and from 88.2 to 94.4% (average: 90.5%), respectively (Table 2). Note that the median is greater than the mean, which indicates that the recall distribution is asymmetric based on the mean but left skewed (negative skewness), meaning that poor performance is infrequent (Figure 10), showing that the SCDA can detect a given actual stroma with a probability of 87.1% (mean based) or 90.5% (median based). Furthermore, the mean and median values of precision ranged from 71.3 to 92.1% (average: 84.3%) and from 82.4 to 93.8% (average: 89.7%), respectively, with the left-skewed precision distribution. From the precision result, the probability of stromata detected by the SCDA to human-scored stromata was 84.3% (mean based) or 89.7% (median based). The performance degradation compared with that of precision results from Sample A, which was the image of relatively lower quality than other sample images. Particularly, image A included many blurred blocks due to focusing problems while it was being collected. This issue will be considered in future work. As a result, mean and median values of Dice coefficient values ranged from 75.0 to 91.0% (83.9%) and from 84.5 to 92.6% (average: 88.3%), respectively.

**Table 2:** Summary of the mean and median of precision, recall, and Dice coefficients (%) upon comparing the stromata contour detection algorithm (SCDA) to human raters A and B for six leaf sample images.

	**Comparison 1 (vs. human rater A)**		**Comparison 2 (vs. human rater B)**	
	**Mean**	**Median**	**Mean**	**Median**
**Sample A**				
Recall (%)	89.0	90.6	88.9	90.8
Precision (%)	71.4	84.0	71.3	82.4
Dice (%)	75.0	84.5	75.2	84.6
**Sample B**				
Recall (%)	85.9	88.2	84.3	86.4
Precision (%)	79.3	86.3	80.1	86.6
Dice (%)	81.0	86.0	80.6	85.0
**Sample C**				
Recall (%)	91.7	94.4	85.7	92.1
Precision (%)	91.6	93.5	89.2	91.7
Dice (%)	91.0	92.6	87.6	89.8
**Sample D**				
Recall (%)	89.0	90.4	87.9	89.4
Precision (%)	91.5	93.8	88.8	91.4
Dice (%)	89.8	91.3	87.8	89.5
**Sample E**				
Recall (%)	87.6	91.9	83.3	89.1
Precision (%)	92.1	93.3	89.9	91.9
Dice (%)	88.8	91.5	86.3	89.4
**Sample F**				
Recall (%)	88.1	92.3	83.5	90.0
Precision (%)	84.3	92.2	82.5	89.3
Dice (%)	83.0	89.8	80.3	85.7
	**Recall (%)**	**Precision (%)**	**Dice (%)**	
Average of mean	87.1	84.3	83.9	
Average of median	90.5	89.7	88.3	

### Correlation of AUDPC of Visual Severity, SCDA, and Mask R-CNN

**Figure 11:**
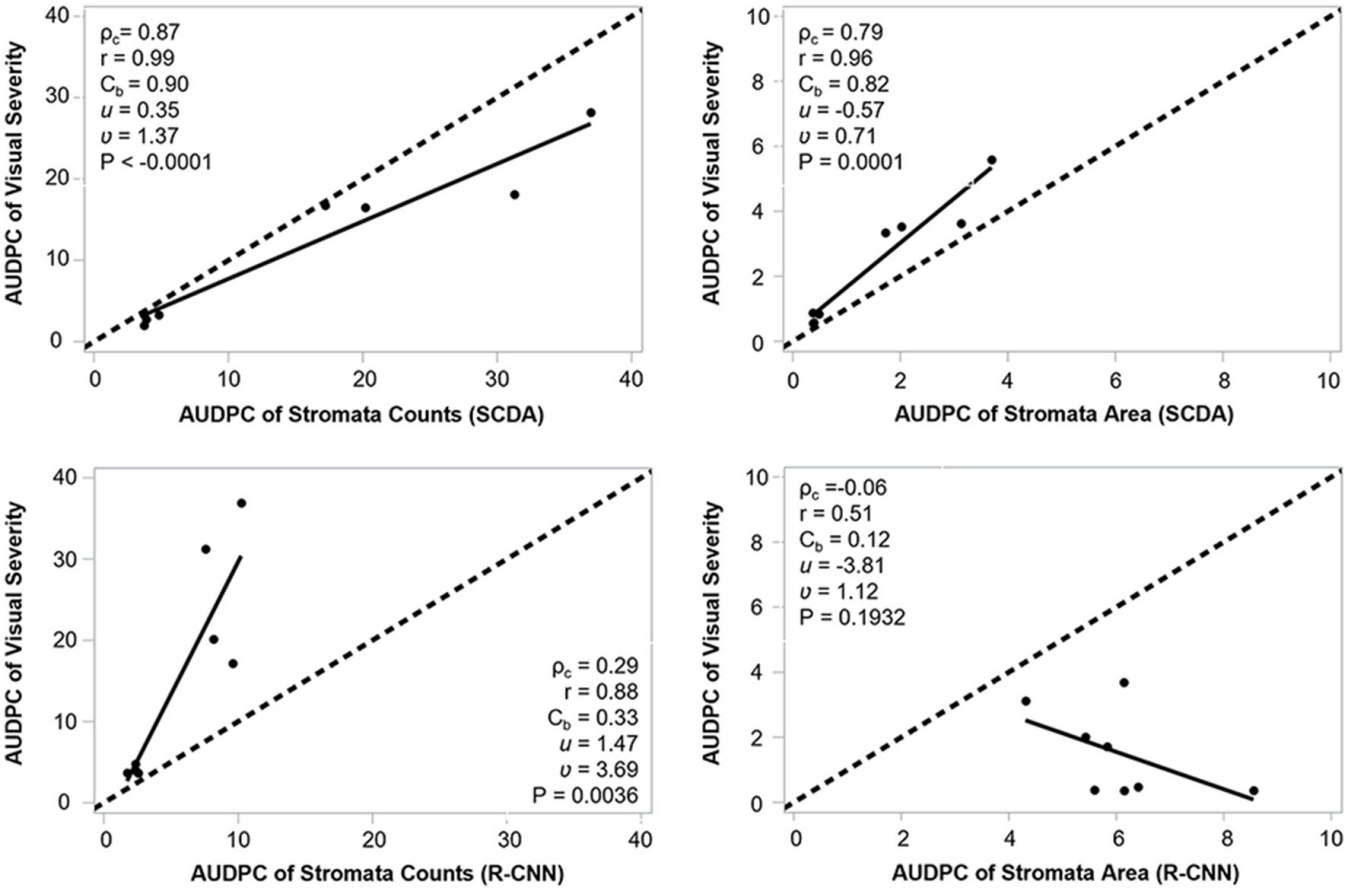
Agreement of AUDPC between visual disease severity vs stromata counts and proportion of leaf covered by stromata generated by SCDA and mask R-CNN.

We observed a higher agreement between AUDPC of visual severity and AUDPC of SCDA at the three canopy levels (ρ_*c*_ = 0.75, r = 0.82, *C*_*b*_ = 0.82) than AUDPC from the R-CNN model (ρ_*c*_ = 0.14, r = 0.13, *C*_*b*_ = 0.27). In general, AUDPC from stromata counts (ρ_*c*_ = 0.82, r = 0.87, *C*_*b*_ = 0.95) had better correlation with AUDPC from the visual estimation than the AUDPC from the area occupied by the stromata (ρ_*c*_ = 0.60, r = 0.87, *C*_*b*_ = 0.69). The best correlation occurred at the mid and upper canopy between AUDPC from visual and AUDPC from the counts of stromata (Figure 11; Table 3).

**Table 3:** Correlation of AUDPC between visual disease severity vs. SCDA and mask R-CNN, respectively, at lower, middle, and upper canopies of the experimental plots.

**Comparison**	**Canopy level**	**Parameter**	**CCC**	**r**	***C*_*b*_**	***u***	**ν**
Visual severity vs. SCDA	Lower	Area_AUDPC	0.51	0.72	0.72	−0.80	0.69
		Count_AUDPC	0.70	0.72	0.96	0.06	1.33
	Middle	Area_AUDPC	0.79	0.96	0.82	−0.57	0.71
		Count_AUDPC	0.87	0.97	0.90	0.35	1.37
	Upper	Area_AUDPC	0.49	0.92	0.53	−1.32	0.51
		Count_AUDPC	0.91	0.93	0.98	−0.22	0.98
	Total	Area_AUDPC	0.59	0.84	0.71	−0.80	0.65
		Count_AUDPC	0.82	0.84	0.97	0.08	1.26
Visual severity vs. R-CNN	Lower	Area_AUDPC	−0.06	−0.59	0.10	−4.14	1.09
		Count_AUDPC	0.27	0.65	0.41	1.00	3.58
	Middle	Area_AUDPC	−0.06	−0.51	0.12	−3.81	1.12
		Count_AUDPC	0.29	0.88	0.33	1.47	3.69
	Upper	Area_AUDPC	−0.03	−0.48	0.07	−5.07	0.80
		Count_AUDPC	0.47	0.82	0.57	0.69	2.66
	Total	Area_AUDPC	−0.05	−0.52	0.10	−4.23	1.03
		Count_AUDPC	0.31	0.76	0.41	1.06	3.41

## Discussion

A successful automated system for disease assessment should provide reproducible results and approach the accuracies achieved by human performance. The outcomes of our study suggest the potential of RGB image processing using contour analysis to mimic human rater assessments of tar spot stromata counts on leaves. The SCDA performed with high accuracy and reliability in quantifying the number of stromata to assess the disease intensity and detect “true stromata” as recognized by human raters. Moreover, we observed that stromata detected by the SCDA were highly correlated with reference ground truth recognized by human raters. Accurate numerical descriptions of the extent of manifestations (symptoms) brought about by the disease and of pathogen structures (signs) observed in a diseased plant sample are essential to correctly assess the effect of the disease and to devise effective management strategies (Nutter and Schultz, 1995; Nutter et al., 2006). Although software tools have been used to quantify the disease severity (Biernacki and Bruton, 2001; Stewart and McDonald, 2014; Rivera et al., 2020), they are neither efficient nor appropriate for the disease count task discussed here. To our knowledge, RGB image-based quantification for tar spot of corn has not been established previously. Since counting thousands of stromata present on leaf images or leaf samples is tedious and labor-intensive, the resulting fatigue can lead to inaccurate counts of stromata, providing less reliable data. To prevent such situations, we utilized Fiji (ImageJ) in this study, which allowed for increased accuracy of detection based on the recognition of the stromata by human raters. This prevented the raters from counting the same stroma more than once and allowed for the tracking of the stromata locations on each leaf.

The generation of image blocks or partitioning single images into equal sizes used these “blocks” for the downstream analysis. The rationale for this procedure was that image blocks decrease the computational cost required for analyzing an entire leaf sample, they increase the number of samples (i.e., image blocks). In addition, this approach also reduces rater subjectivity, which is crucial as the labor-intensive nature of generating ground truth data affects the reliability and accuracy of the results (Bock et al., 2010). Moreover, by partitioning the whole leaf into image blocks, a wide variation in disease intensity (stromata count) can be analyzed.

The under-or over-estimation of the SCDA may be accounted for by its limitation to detect small-sized stromata and the low resolution in regions beyond the focus of the camera. The use of flatbed scanners is one of our recommendations to address this issue, although obtaining high-resolution images may take a bit more time than using a camera. Moreover, the noise was eliminated in our image blocks using a Gaussian filter to generate a high-quality image before processing each image for feature extraction. Images were enhanced by utilizing a Gaussian filter, which blurs the images by suppressing high frequencies, similar to the effect of the mean filter. The Gaussian filter has been used previously in image-based plant disease detection (Camargo and Smith, 2009; Shrivastava et al., 2017).

In some cases, another type of manifestation of tar spot is “fish-eye” symptoms, which often appear after stromata structures have emerged and are visible (Hock et al., 1992, 1995; Bajet et al., 1994). These lesions are characterized by *Phyllachora maydis* stromata at their centers while surrounded by ellipsoidal, chlorotic/necrotic halos, which can enhance the severity of tar spots (Hock et al., 1992; Bajet et al., 1994). Detecting these types of symptoms was not the scope of this study as they are not as common in northern North America; however, this is a recommended enhancement for future research projects. In addition, the condition of the leaf samples was preserved by using a leaf press and storing them at 4°C. Along with the stromata contour detection algorithm (SCDA) in providing accurate disease intensity quantification in the lab, field disease evaluations still need improvement.

For the optimal performance of the proposed algorithm, one should set parameters properly, such as (1) the number of smaller sub-contour lines surrounding a stroma (*N*_*ct*_), (2) the degree of roundness (*g*_*circle*_), and (3) the ratio of areas of a pair of nearest sub-contour lines ($r_{ad}=\frac{area\;of\;the\;smaller\;contour}{area\;of\;the\;larger\;contour}\leq 1$). Moreover, for a given image block, the determination of these parameters mainly depends on (1) the blurriness of the grayscale image and (2) the total number of contour levels used to discretize a grayscale level of the image block. In principle, the optimal parameters may be different for each image block, even on the same corn leaf, since the extents of the intensity in homogeneities and blurriness due to a focusing spot imposed on a raw RGB image are different in general. That is why we applied the two-step preprocessing (i.e., intensity homogenization and Gaussian filtering) to make sure that all image blocks would be in a similar condition as far as possible. Consequently, the same parameters used in analyzing all image blocks may not lead to significant degradation.

Nevertheless, when an image block is too blurry due to the Gaussian filter with a larger window, smaller tar spot stromata tend to be wiped out along with salt-and-pepper noise; thus, they are never recognized. On the other hand, analyzing an image block that is less blurred requires a more significant number of contour levels to detect smaller tar spot stromata, ending up with expensive computational costs. Specifically, when more contour levels are used for a less blurred image block, contour lines around a tar spot stroma tend to be more densely populated; thus, *N*_*ct*_ generally increases, and *r*_*ad*_ converges to unity allowing for a higher probability of detecting true-positive cases since the detection of stromata may not be very sensitive to the parameters chosen. However, when insufficient numbers of contour levels are used for a less blurred image block, the performance may degrade significantly; specifically, many false-positive cases may occur. In contrast, for an image block that is blurred excessively, the performance would be saturated even with the use of numerous contour levels. This is because information on smaller tar spot stromata was already lost during the course of blurring. As a consequence, a tradeoff exists between computational efficiency and accuracy. Therefore, the accuracy is determined by the degree of blurriness applied to an image block. Specifically, it determines the smallest size of tar spot stromata that can be found. Then, one can find an optimal number of contour levels for the best performance in detecting tar spot stromata for the blurred image block, which determines computational costs.

In general, both SCDA and mask R-CNN were able to detect and measure the number of stromata and diseased areas covered by the stromata. However, SCDA performed better than the mask R-CNN algorithm based on its correlation of estimated visual severity (AUDPC). Both SCDA and mask R-CNN separated plots that were controls (untreated plots) from those used as treatments (with fungicide application). Generally, control plots had higher values for visual tar spot disease severity, stromata counts, and leaf area covered with stromata. However, the SCDA showed a significantly greater area under disease progress curve (AUDPC) agreement with visual disease estimations compared to mask R-CNN. For future research, we can employ artificial intelligence to train a machine to automatically find the optimal parameters for a given image block while performing the contour-based stromata detection analysis. This is beyond the scope of the present project.

In our study, we utilized a laptop with Intel Core i7-8650U processor (with an 8-MB cache memory and a base frequency of 1.9 GHz and a maximum frequency of 4.2 GHz) and 16 GB of RAM for the present analyses. For our empirical observation with several trials and errors, we found that it would be the best setup with 100 contour levels for each image block and σ = 2 and σ = 1.5 for coarse and fine windows, respectively, for using the Gaussian filter provided by MATLAB. Note that σ represents a standard deviation of the two-dimensional Gaussian distribution. With this parameter setup, we set *N*_*ct*_ = 10 and *N*_*ct*_ = 5 in searching stromata in coarse and fine windows, respectively. For both windows, we set *g*_*circle*_ = 0.25 and *r*_*ad*_ = 0.7. It is worth noting that the reason why *g*_*circle*_ was set to a relatively lower value is because of the detection of matured tar spot stromata that tend to form an ellipse-like shape.

Plant disease intensity is often measured with random variables. In some instances, pathogen density based on the number of stromata per unit leaf area may be a better measure of disease intensity than the visual severity in terms of inter-rater repeatability (Madden et al., 2007). Also, severity and counts are different concepts from a statistical standpoint. The count is a discrete variable, and severity is a continuous, random variable. Although discrete stromata count data can encapsulate and convey the natural progression of pathogen invasion and disease development, without automation, counting stromata is time-consuming and tiring. Automated counting of physically distinct stromata is an option with plant diseases as characteristic and conspicuous as tar spot of corn. Automated disease measurement is still in an exploratory stage and the results presented are the basis of future research based on data collected under field conditions and data processing with more advanced techniques. Our ultimate goal is to explore the spatio-temporal domain of plant disease quantification using both visual and digital imagery and weather variables to properly describe and forecast plant disease epidemics.

## Conclusion

Automated, image-based, accurate detection and assessment of disease intensity will provide a substitute for labor-intensive and subjective-prone, human visual-based disease intensity estimations and aid in generating high volumes of reliable data in a relatively short time. In turn, this will support building robust epidemiological models for tar spot outbreaks and improving the management decisions for this disease. Moreover, for an emerging disease, such as tar spot, it is crucial to develop and establish a standardized method that will provide accurate estimates of plant disease intensity to obtain reliable assessments for monitoring tar spot epidemics, resistance screening, and management practices. The contour-based stromata detection method developed in this study will serve as a foundation toward building a systematic approach in quantifying the disease intensity of tar spot using digital imagery as well as for other plant diseases generating similar types of stromata.

## Data Availability Statement

The original contributions presented in the study are publicly available. This data can be found here: https://purr.purdue.edu/publications/3820/2. Further inquiries can be directed to the corresponding author.

## Author Contributions

D-YL and CC conceived the idea. D-YL and D-YN designed and developed the stromata contour detection algorithm. SB developed the mask R-CNN. D-YL, D-YN, and CG-C performed statistical data analyses and interpretation. BL, AC, and MF-C provided human visual-based disease assessments. D-YL and D-YN wrote the manuscript with significant contributions by CG-C and SB. NK and DT provided the experiment site. NK, DT, ED, SG, and CC reviewed the manuscript. All authors contributed to the article and approved the submitted version.

## Funding

This work was supported by Purdue University as part of the Agriculture-Engineering initiative aimed at strengthening interdisciplinary collaboration between the Colleges of Agriculture and Engineering, Indiana Corn and Marketing Council (ICMC; grant number 40003362), and United States Department of Agriculture, Agricultural Research Service (USDA-ARS) research project 5020-21220-019-016-S.

## Conflict of Interest

The authors declare that the research was conducted in the absence of any commercial or financial relationships that could be construed as a potential conflict of interest.

## Publisher's Note

All claims expressed in this article are solely those of the authors and do not necessarily represent those of their affiliated organizations, or those of the publisher, the editors and the reviewers. Any product that may be evaluated in this article, or claim that may be made by its manufacturer, is not guaranteed or endorsed by the publisher.
